# The genome sequence of spotted medick, *Medicago arabi*ca (L.) Huds. (Fabaceae)

**DOI:** 10.12688/wellcomeopenres.20996.2

**Published:** 2024-11-04

**Authors:** Maarten J. M. Christenhusz, Michael F. Fay, Ilia J. Leitch, Royal Botanic Gardens Kew Genome Acquisition Lab, Plant Genome Sizing collective, Darwin Tree of Life Barcoding collective, Wellcome Sanger Institute Tree of Life Management, Samples and Laboratory team, Wellcome Sanger Institute Scientific Operations: Sequencing Operations, Wellcome Sanger Institute Tree of Life Core Informatics team, Tree of Life Core Informatics collective, Darwin Tree of Life Consortium

**Affiliations:** 1 Royal Botanic Gardens Kew, Richmond, England, UK; 2 Curtin University, Perth, Western Australia, Australia; 3 The University of Western Australia, Perth, Western Australia, Australia

**Keywords:** Medicago arabica, spotted medick, genome sequence, chromosomal, Fabales

## Abstract

We present a genome assembly from an individual *Medicago arabica* (the spotted medick; Tracheophyta; Magnoliopsida; Fabales; Fabaceae). The genome sequence is 515.5 megabases in span. Most of the assembly is scaffolded into 8 chromosomal pseudomolecules. The mitochondrial and plastid genome assemblies have lengths of 324.47 kilobases and 125.07 kilobases in length, respectively. Gene annotation of this assembly on Ensembl identified 24,619 protein-coding genes.

## Species taxonomy

Eukaryota; Viridiplantae; Streptophyta; Streptophytina; Embryophyta; Tracheophyta; Euphyllophyta; Spermatophyta; Magnoliopsida; Mesangiospermae; eudicotyledons; Gunneridae; Pentapetalae; rosids; fabids; Fabales; Fabaceae; Papilionoideae; 50 kb inversion clade; NPAAA clade; Hologalegina; IRL clade; Trifolieae; *Medicago*; *Medicago arabica* (L.) Huds. (NCBI:txid70936).

## Background

Spotted medick, *Medicago arabica* (L.) Huds., is a winter-growing annual of the pea family, Fabaceae. It has creeping stems bearing trifoliate leaves, with each leaflet marked with dark purple spots. Yellow flowers appear in spring and early summer, followed by coiled, spiny seed pods that stick in the fur of animals (and clothes of humans), aiding their dispersal.

The species is native to the Mediterranean region, east to the Caucasus and Crimea, and is found along the Atlantic in western Europe, and north to Britain. It is frequently naturalised as a wool alien outside its natural range (e.g. in USA, Costa Rica, southern South America, Japan, China, Australia, New Zealand, Alps, Baltic States, Sweden, Ireland). In Britain, it has a predominantly southern and south-eastern distribution (e.g. Botanical Society of Britain and Ireland, 2024), being most common in southern England to the Midlands; it is much rarer in Wales and northern England and Scotland, where it occurs mostly along the coast. In England it is increasingly found in inland, lowland areas, but for reasons that are not known ( OABIF, 2022; POWO, 2024). It grows in grassy places usually on light soils, and it can be found as a weed in lawns and in fields, roadside verges and rough ground.

Comparative genomics of *M. arabica*, *M. sativa* and other *Medicago* species could provide useful information on traits with agronomic potential for plant breeders. Like many other *Medicago* species, spotted medick is rich in a variety of saponins, with potential applications as antimicrobial compounds in agriculture and medicine (e.g. Avato *et al.,* 2006; Bialy *et al*., 2004; Jarecka *et al.*, 2008; Tava *et al*., 2009).

*Medicago arabica* is a diploid, with 16 chromosomes (e.g. Fyad-Lameche *et al*., 2016). It is a relative of the important forage crop lucerne (alfalfa; *Medicago sativa* L.), which is a tetraploid (2 *n* = 32). Like many other *Medicago* species, spotted medick is rich in a variety of saponins with potential for use as antimicrobial compounds in agriculture and medicine (e.g. Avato *et al*., 2006; Bialy *et al*., 2004; Jarecka *et al*., 2008; Tava *et al*., 2009).

Here we present the first high-quality genome of *Medicago arabica* which will not only help shed light on the biochemical pathways involved in the biosynthesis of saponins, but may also be useful for comparative genomic studies with cultivated *Medicago* species and their wild relatives, providing useful information on traits of agronomic potential for plant breeders. For example, it joins the chromosome level genome assemblies available for three agriculturally important *Medicago* species comprising (i) alfalfa, *M. sativa* ( Chen *et al.*, 2020) which is globally one of the highest yielding forage crops; (ii) the bur clover, *M. polymorpha* ( Cui *et al.*, 2021), cultivated for its low lignin content which makes it particularly nutritious; and (iii) *M. ruthenica* ( Wang *et al.*, 2021), a wild relative of *M. sativa* that is tolerant of environmental stress.

## Genome sequence report

**Figure 1.**
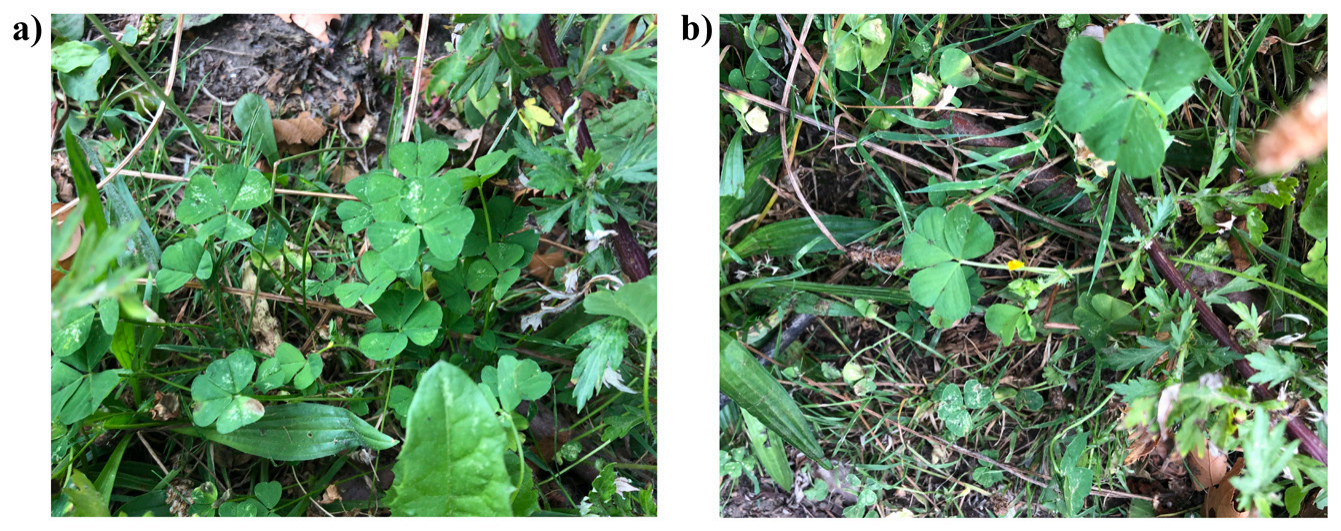
Photographs of the *Medicago arabica* (drMedArab1) specimen used for genome sequencing.

The genome was sequenced from a specimen of *Medicago arabica* ( Figure 1) collected from Kingston Upon Thames, Surrey, UK (51.42, –0.31). Using flow cytometry, the genome size (1C-value) was estimated to be 0.62 pg, equivalent to 610 Mb. A total of 44-fold coverage in Pacific Biosciences single-molecule HiFi long reads and 86-fold coverage in 10X Genomics read clouds was generated. Primary assembly contigs were scaffolded with chromosome conformation Hi-C data. Manual assembly curation corrected 106 missing joins or mis-joins and removed 2 haplotypic duplications, reducing the scaffold number by 33.13%, and increasing the scaffold N50 by 24.91%.

**Figure 2.**
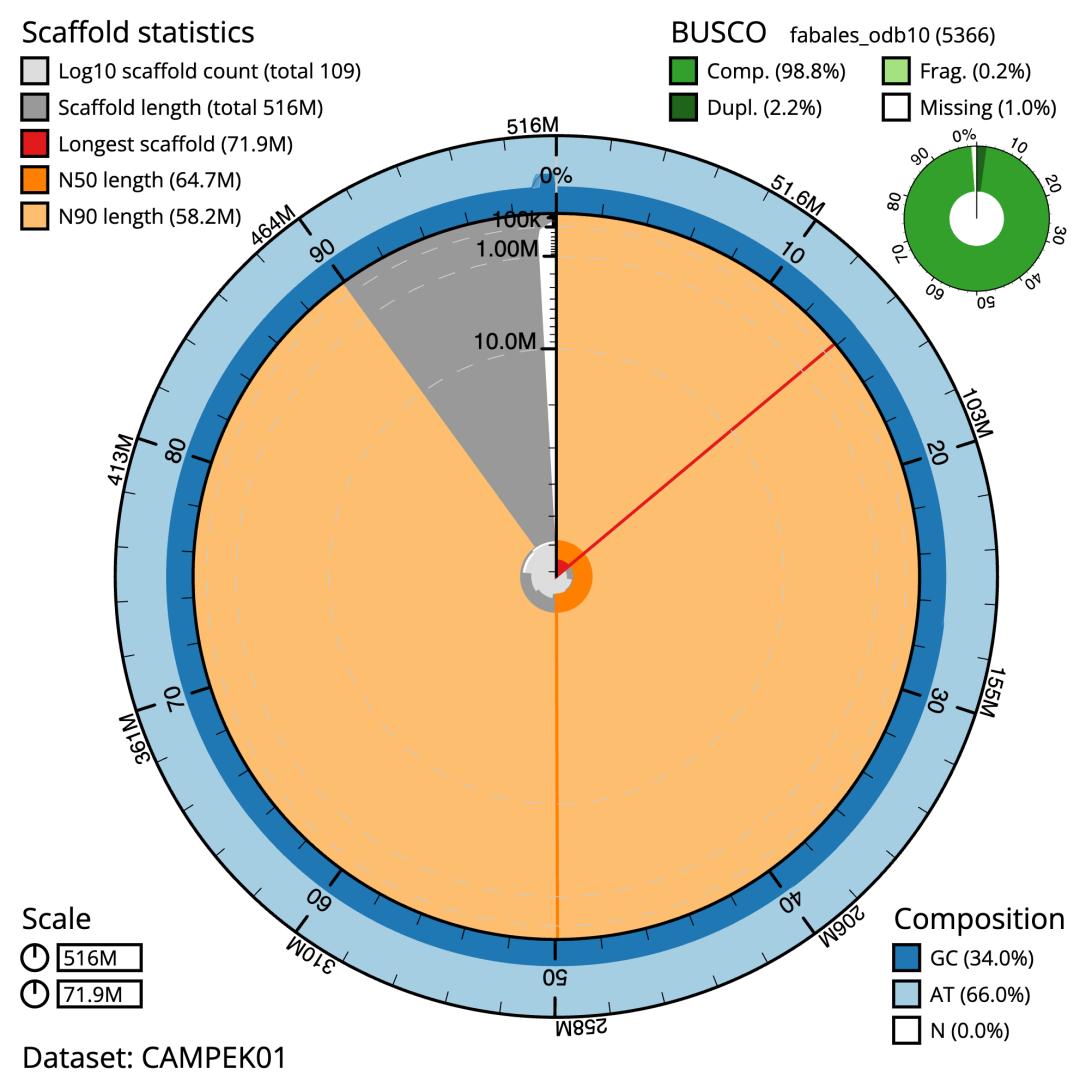
Genome assembly of *Medicago arabica*, drMedArab1.1: metrics. The BlobToolKit Snailplot shows N50 metrics and BUSCO gene completeness. The main plot is divided into 1,000 bins around the circumference with each bin representing 0.1% of the 515,954,536 bp assembly. The distribution of scaffold lengths is shown in dark grey with the plot radius scaled to the longest scaffold present in the assembly (71,875,296 bp, shown in red). Orange and pale-orange arcs show the N50 and N90 scaffold lengths (64,674,077 and 58,228,340 bp), respectively. The pale grey spiral shows the cumulative scaffold count on a log scale with white scale lines showing successive orders of magnitude. The blue and pale-blue area around the outside of the plot shows the distribution of GC, AT and N percentages in the same bins as the inner plot. A summary of complete, fragmented, duplicated and missing BUSCO genes in the fabales_odb10 set is shown in the top right. An interactive version of this figure is available at https://blobtoolkit.genomehubs.org/view/CAMPEK01/dataset/CAMPEK01/snail.

**Figure 3.**
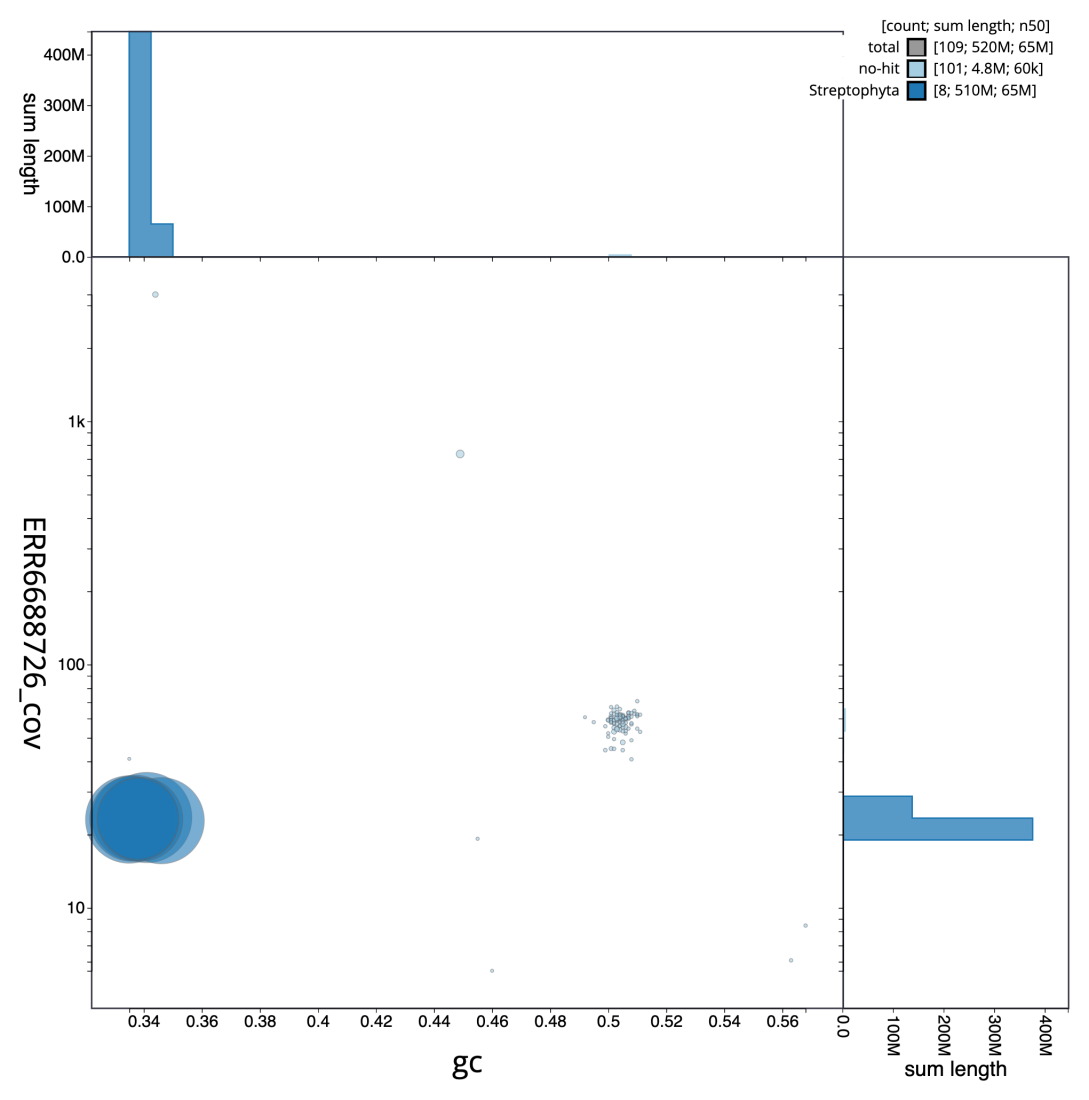
Genome assembly of *Medicago arabica*, drMedArab1.1: BlobToolKit GC-coverage plot. Scaffolds are coloured by phylum. Circles are sized in proportion to scaffold length. Histograms show the distribution of scaffold length sum along each axis. An interactive version of this figure is available at https://blobtoolkit.genomehubs.org/view/CAMPEK01/dataset/CAMPEK01/blob.

**Figure 4.**
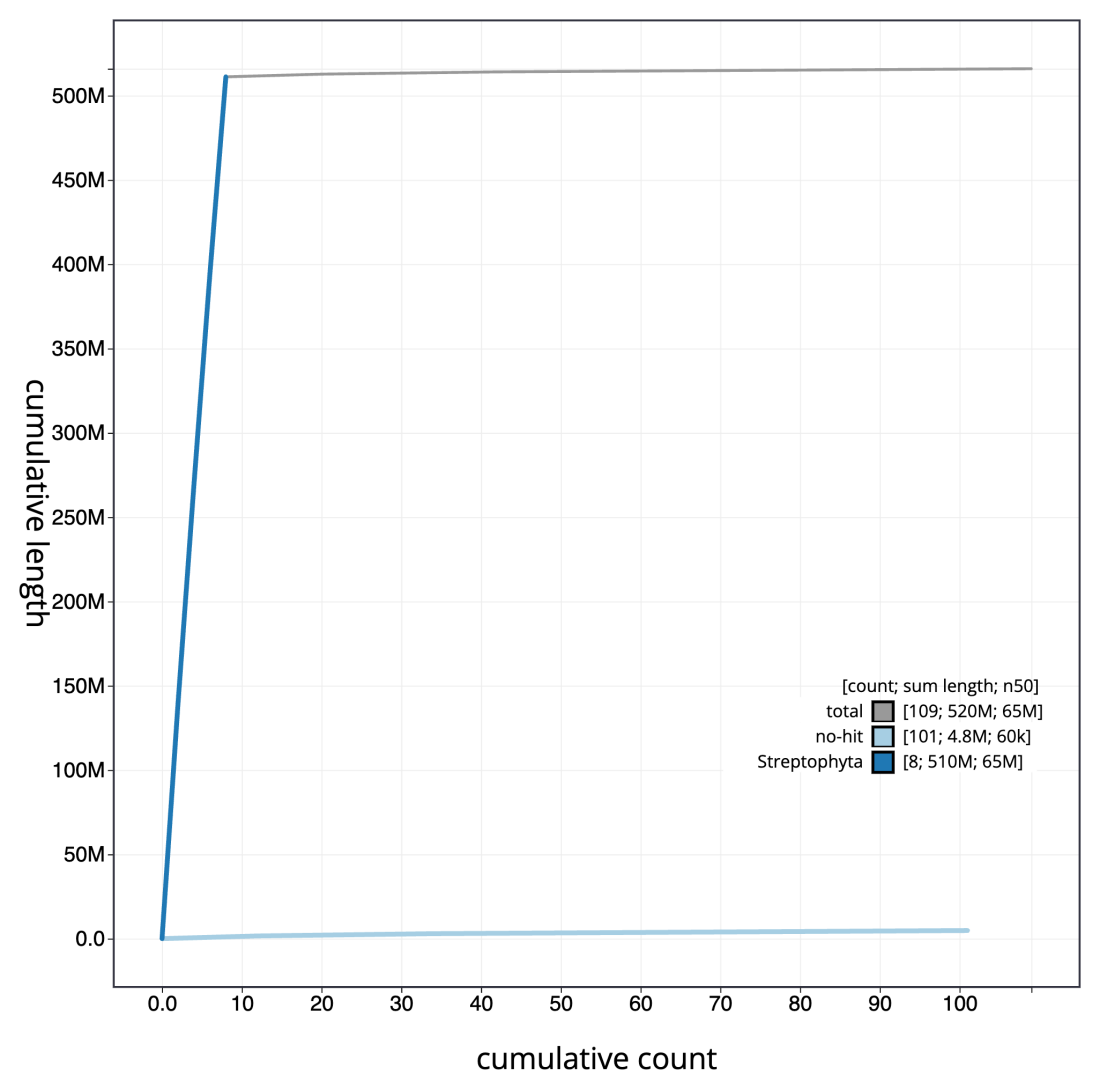
Genome assembly of *Medicago arabica*, drMedArab1.1: BlobToolKit cumulative sequence plot. The grey line shows cumulative length for all scaffolds. Coloured lines show cumulative lengths of scaffolds assigned to each phylum using the buscogenes taxrule. An interactive version of this figure is available at https://blobtoolkit.genomehubs.org/view/CAMPEK01/dataset/CAMPEK01/cumulative.

**Figure 5.**
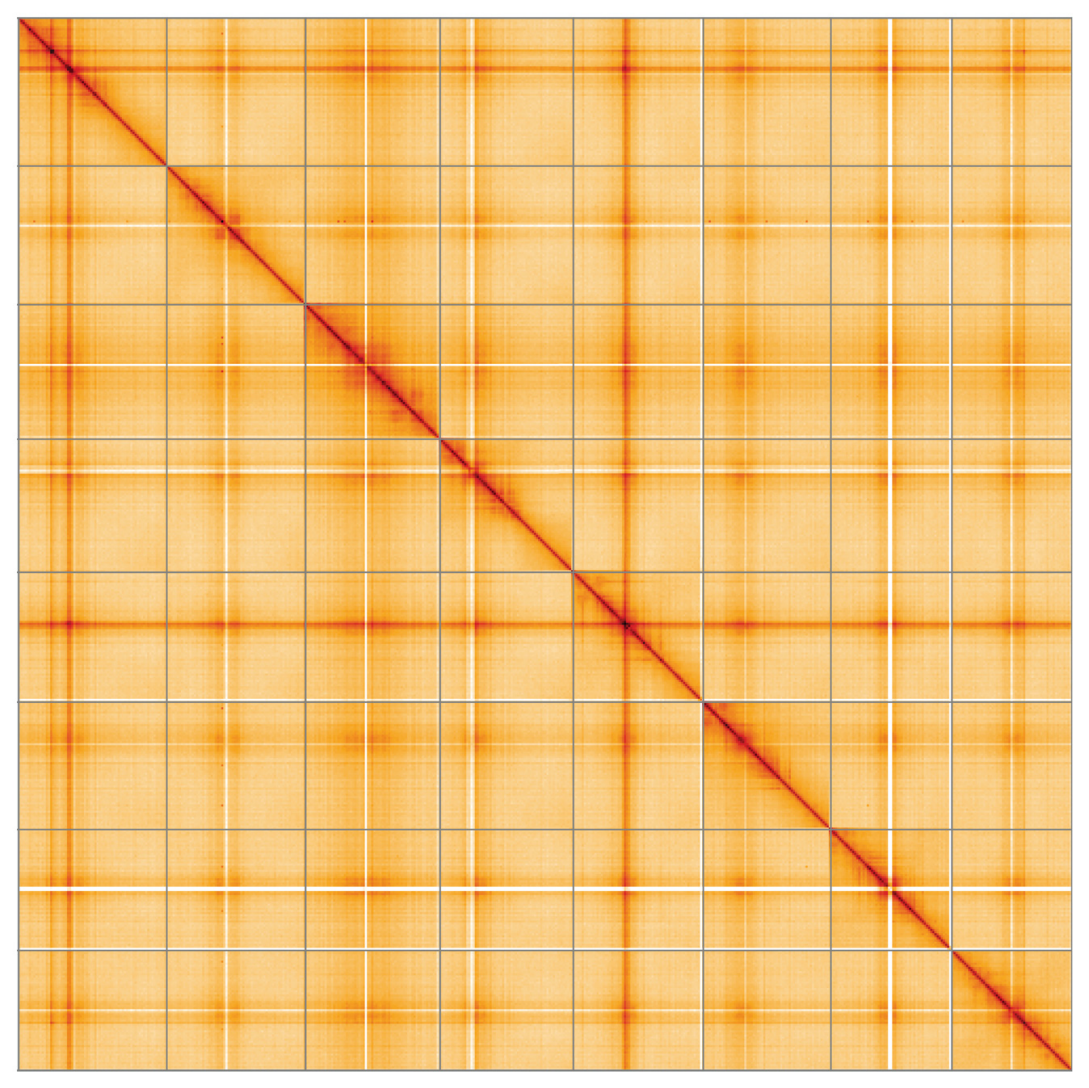
Genome assembly of *Medicago arabica*, drMedArab1.1: Hi-C contact map of the drMedArab1.1 assembly, visualised using HiGlass. Chromosomes are shown in order of size from left to right and top to bottom. An interactive version of this figure may be viewed at https://genome-note-higlass.tol.sanger.ac.uk/l/?d=FKwiULypRCm3Y-gUQXShkQ.

The final assembly has a total length of 515.5 Mb in 107 sequence scaffolds with a scaffold N50 of 64.7 Mb ( Table 1). The snail plot in Figure 2 provides a summary of the assembly statistics, while the distribution of assembly scaffolds on GC proportion and coverage is shown in Figure 3. The cumulative assembly plot in Figure 4 shows curves for subsets of scaffolds assigned to different phyla. Most (99.91%) of the assembly sequence was assigned to 8 chromosomal-level scaffolds. Chromosome-scale scaffolds confirmed by the Hi-C data are named in order of size ( Figure 5; Table 2). Parts of the rRNA cluster on chromosome 1 at 24.5Mbp could not be uniquely placed and were submitted as unlocalised sequences of chromosome 1. While not fully phased, the assembly deposited is of one haplotype. Contigs corresponding to the second haplotype have also been deposited. The mitochondrial and plastid genomes were also assembled and can be found as contigs within the multifasta file of the genome submission.

**Table 1.** Genome data for *Medicago arabica*, drMedArab1.1.

Project accession data		
Assembly identifier	drMedArab1.1	
Species	*Medicago arabica*	
Specimen	drMedArab1	
NCBI taxonomy ID	70936	
BioProject	PRJEB47317	
BioSample ID	SAMEA7521936	
Isolate information	drMedArab1	
Assembly metrics *		*Benchmark*
Consensus quality (QV)	56.4	*≥ 40*
*k*-mer completeness	99.99%	*≥ 95%*
BUSCO **	C:98.8%[S:96.6%,D:2.2%], F:0.2%,M:1.0%,n:5,366	*C ≥ 95%*
Percentage of assembly mapped to chromosomes	99.91%	*≥ 90%*
Sex chromosomes	None	*localised homologous pairs*
Organelles	Mitochondrial genome: 324.47 kb Plastid genome: 125.07 kb	*complete single alleles*
Raw data accessions		
PacificBiosciences SEQUEL II	ERR6908000	
10X Genomics Illumina	ERR6688727, ERR6688725, ERR6688726, ERR6688728	
Hi-C Illumina	ERR6688404	
PolyA RNA-Seq Illumina	ERR6688729	
Genome assembly		
Assembly accession	GCA_946800305.1	
*Accession of alternate haplotype*	GCA_946800295.1	
Span (Mb)	515.5	
Number of contigs	235	
Contig N50 length (Mb)	6.5	
Number of scaffolds	107	
Scaffold N50 length (Mb)	64.7	
Longest scaffold (Mb)	76.24	
Genome annotation of assembly GCA_946800305.1 at Ensembl		
Number of protein-coding genes	24,619	
Number of non-coding genes	8,254	
Number of gene transcripts	40,979	

* Assembly metric benchmarks are adapted from column VGP-2020 of “Table 1: Proposed standards and metrics for defining genome assembly quality” from Rhie *et al.* (2021). ** BUSCO scores based on the fabales_odb10 BUSCO set using version 5.3.2. C = complete [S = single copy, D = duplicated], F = fragmented, M = missing, n = number of orthologues in comparison. A full set of BUSCO scores is available at https://blobtoolkit.genomehubs.org/view/CAMPEK01/dataset/CAMPEK01/busco.

**Table 2.** Chromosomal pseudomolecules in the genome assembly of *Medicago arabica*, drMedArab1.

INSDC accession	Chromosome	Length (Mb)	GC%
OX326964.1	1	71.88	34.0
OX326965.1	2	67.34	33.5
OX326966.1	3	65.33	34.5
OX326967.1	4	64.67	33.5
OX326968.1	5	63.06	33.5
OX326969.1	6	61.9	34.0
OX326970.1	7	58.74	34.0
OX326971.1	8	58.23	34.0
OX326972.1	MT	0.32	45.0
OX326973.1	Pltd	0.13	34.5

## Genome annotation report

The Medicago arabica genome assembly (GCA_946800305.1) was annotated at the European Bioinformatics Institute (EBI) on Ensembl Rapid Release. The resulting annotation includes 40,979 transcribed mRNAs from 24,619 protein-coding and 8,254 non-coding genes ( Table 2; https://rapid.ensembl.org/Medicago_arabica_GCA_946800305.1/Info/Index). The average transcript length is 2,758.62. There are 1.25 coding transcripts per gene and 4.52 exons per transcript.

The estimated Quality Value (QV) of the final assembly is 56.4 with *k*-mer completeness of 99.99%, and the assembly has a BUSCO v5.3.2 completeness of 98.8% (single = 96.6%, duplicated = 2.2%), using the fabales_odb10 reference set ( *n* = 5,366).

Metadata for specimens, barcode results, spectra estimates, sequencing runs, contaminants and pre-curation assembly statistics are given at https://links.tol.sanger.ac.uk/species/70936.

## Methods

### Sample acquisition, genome size estimation and nucleic acid extraction

A specimen of *Medicago arabica* (specimen ID KDTOL10027, ToLID drMedArab1) was collected from Canbury Gardens, Kingston Upon Thames, Surrey, UK (latitude 51.42, longitude –0.31) on 2020-08-10. The specimen was collected and identified by Maarten Christenhusz (Royal Botanic Gardens Kew), and then preserved by freezing at –80 °C.

The genome size was estimated by flow cytometry using the fluorochrome propidium iodide and following the ‘one-step’ method as outlined in Pellicer *et al.* (2021). For this species, the General Purpose Buffer (GPB) supplemented with 3% PVP and 0.08% (v/v) beta-mercaptoethanol was used for isolation of nuclei ( Loureiro *et al.*, 2007), and the internal calibration standard was *Solanum lycopersicum* ‘Stupiké polní rané’ with an assumed 1C-value of 968 Mb ( Dolezel *et al.*, 2007).

The workflow for high molecular weight (HMW) DNA extraction at the Wellcome Sanger Institute (WSI) includes a sequence of core procedures: sample preparation; sample homogenisation, DNA extraction, fragmentation, and clean-up. In sample preparation, the drMedArab1 sample was weighed and dissected on dry ice ( Jay *et al.*, 2023). For sample homogenisation, leaf tissue was cryogenically disrupted using the Covaris cryoPREP ^®^ Automated Dry Pulverizer ( Narváez-Gómez *et al.*, 2023). HMW DNA was extracted using the Manual Plant MagAttract v2 protocol ( Todorovic *et al.*, 2023a). HMW DNA was sheared into an average fragment size of 12–20 kb in a Megaruptor 3 system with speed setting 30 ( Todorovic *et al.*, 2023b). Sheared DNA was purified by solid-phase reversible immobilisation ( Strickland *et al.*, 2023): in brief, the method employs a 1.8X ratio of AMPure PB beads to sample to eliminate shorter fragments and concentrate the DNA. The concentration of the sheared and purified DNA was assessed using a Nanodrop spectrophotometer and Qubit Fluorometer and Qubit dsDNA High Sensitivity Assay kit. Fragment size distribution was evaluated by running the sample on the FemtoPulse system.

RNA was extracted from leaf tissue of drMedArab1 in the Tree of Life Laboratory at the WSI using the RNA Extraction: Automated MagMax™ *mir*Vana protocol ( do Amaral *et al.*, 2023). The RNA concentration was assessed using a Nanodrop spectrophotometer and a Qubit Fluorometer using the Qubit RNA Broad-Range Assay kit. Analysis of the integrity of the RNA was done using the Agilent RNA 6000 Pico Kit and Eukaryotic Total RNA assay.

Protocols developed by the WSI Tree of Life core laboratory are publicly available on protocols.io ( Denton *et al.*, 2023).

### Sequencing

Pacific Biosciences HiFi circular consensus and 10X Genomics read cloud DNA sequencing libraries were constructed according to the manufacturers’ instructions. Poly(A) RNA-Seq libraries were constructed using the NEB Ultra II RNA Library Prep kit. DNA and RNA sequencing was performed by the Scientific Operations core at the WSI on Pacific Biosciences SEQUEL II (HiFi), Illumina HiSeq 4000 (RNA-Seq) and Illumina NovaSeq 6000 (10X) instruments. Hi-C data were also generated from leaf tissue of drMedArab1 using the Arima2 kit and sequenced on the Illumina NovaSeq 6000 instrument.

### Genome assembly, curation and evaluation

Assembly was carried out with Hifiasm ( Cheng *et al.*, 2021) with the --primary option, and the primary contigs were used for the remainder of the assembly pipeline. Haplotypic duplication was identified and removed with purge_dups ( Guan *et al.*, 2020). One round of polishing was performed by aligning 10X Genomics read data to the assembly with Long Ranger ALIGN, calling variants with FreeBayes ( Garrison & Marth, 2012). The assembly was then scaffolded with Hi-C data ( Rao *et al.*, 2014) using SALSA2 ( Ghurye *et al.*, 2019). The assembly was checked for contamination and corrected using the gEVAL system ( Chow *et al.*, 2016) as described previously ( Howe *et al.*, 2021). Manual curation was performed using gEVAL, HiGlass ( Kerpedjiev *et al.*, 2018) and PretextView ( Harry, 2022). The mitochondrial and chloroplast genomes were assembled using MBG ( Rautiainen & Marschall, 2021) from PacBio HiFi reads mapping to related genomes. A representative circular sequence was selected for each from the graph based on read coverage.

A Hi-C map for the final assembly was produced using bwa-mem2 ( Vasimuddin *et al.*, 2019) in the Cooler file format ( Abdennur & Mirny, 2020). To assess the assembly metrics, the *k*-mer completeness and QV consensus quality values were calculated in Merqury. FK ( Rhie *et al.*, 2020). This work was done using Nextflow ( Di Tommaso *et al.*, 2017) DSL2 pipelines “sanger-tol/readmapping” ( Surana *et al.*, 2023a) and “sanger-tol/genomenote” ( Surana *et al.*, 2023b). The genome was analysed within the BlobToolKit environment ( Challis *et al.*, 2020) and BUSCO scores ( Manni *et al.*, 2021; Simão *et al.*, 2015) were calculated.

Table 3 contains a list of relevant software tool versions and sources.

**Table 3.** Software tools: versions and sources.

Software tool	Version	Source
BlobToolKit	3.5.2	https://github.com/blobtoolkit/blobtoolkit
BUSCO	5.3.2	https://gitlab.com/ezlab/busco
FreeBayes	1.3.1-17-gaa2ace8	https://github.com/freebayes/freebayes
gEVAL	N/A	https://geval.org.uk/
Hifiasm	0.15.3	https://github.com/chhylp123/hifiasm
HiGlass	1.11.6	https://github.com/higlass/higlass
Long Ranger ALIGN	2.2.2	https://support.10xgenomics.com/genome-exome/ software/pipelines/latest/advanced/other-pipelines
MBG	1.0.13	https://github.com/maickrau/MBG
Merqury	MerquryFK	https://github.com/thegenemyers/MERQURY.FK
PretextView	0.2	https://github.com/wtsi-hpag/PretextView
purge_dups	1.2.3	https://github.com/dfguan/purge_dups
SALSA	2.2	https://github.com/salsa-rs/salsa
sanger-tol/genomenote	v1.0	https://github.com/sanger-tol/genomenote
sanger-tol/readmapping	1.1.0	https://github.com/sanger-tol/readmapping/tree/1.1.0

### Genome annotation

The Ensembl Genebuild annotation system ( Aken *et al.*, 2016) was used to generate annotation for the *Medicago arabica* assembly (GCA_946800305.1) in Ensembl Rapid Release at the EBI. Annotation was created primarily through alignment of transcriptomic data to the genome, with gap filling via protein-to-genome alignments of a select set of proteins from UniProt ( UniProt Consortium, 2019). 

### Wellcome Sanger Institute – Legal and Governance

The materials that have contributed to this genome note have been supplied by a Darwin Tree of Life Partner. The submission of materials by a Darwin Tree of Life Partner is subject to the **‘Darwin Tree of Life Project Sampling Code of Practice’**, which can be found in full on the Darwin Tree of Life website here. By agreeing with and signing up to the Sampling Code of Practice, the Darwin Tree of Life Partner agrees they will meet the legal and ethical requirements and standards set out within this document in respect of all samples acquired for, and supplied to, the Darwin Tree of Life Project.

Further, the Wellcome Sanger Institute employs a process whereby due diligence is carried out proportionate to the nature of the materials themselves, and the circumstances under which they have been/are to be collected and provided for use. The purpose of this is to address and mitigate any potential legal and/or ethical implications of receipt and use of the materials as part of the research project, and to ensure that in doing so we align with best practice wherever possible. The overarching areas of consideration are:

•      Ethical review of provenance and sourcing of the material

•      Legality of collection, transfer and use (national and international)

Each transfer of samples is further undertaken according to a Research Collaboration Agreement or Material Transfer Agreement entered into by the Darwin Tree of Life Partner, Genome Research Limited (operating as the Wellcome Sanger Institute), and in some circumstances other Darwin Tree of Life collaborators.

## Data Availability

European Nucleotide Archive: *Medicago arabica*. Accession number PRJEB47317; https://identifiers.org/ena.embl/PRJEB47317 ( Wellcome Sanger Institute, 2022). The genome sequence is released openly for reuse. The *Medicago arabica* genome sequencing initiative is part of the Darwin Tree of Life (DToL) project. All raw sequence data and the assembly have been deposited in INSDC databases. The genome will be annotated using available RNA-Seq data and presented through the Ensembl pipeline at the European Bioinformatics Institute. Raw data and assembly accession identifiers are reported in Table 1.

## Author information

Members of the Royal Botanic Gardens Kew Genome Acquisition Lab are listed here: https://doi.org/10.5281/zenodo.4786680.

Members of the Plant Genome Sizing collective are listed here: https://doi.org/10.5281/zenodo.7994306.

Members of the Darwin Tree of Life Barcoding collective are listed here: https://doi.org/10.5281/zenodo.4893703.

Members of the Wellcome Sanger Institute Tree of Life Management, Samples and Laboratory team are listed here: https://doi.org/10.5281/zenodo.10066175.

Members of Wellcome Sanger Institute Scientific Operations: Sequencing Operations are listed here: https://doi.org/10.5281/zenodo.10043364.

Members of the Wellcome Sanger Institute Tree of Life Core Informatics team are listed here: https://doi.org/10.5281/zenodo.10066637.

Members of the Tree of Life Core Informatics collective are listed here: https://doi.org/10.5281/zenodo.5013541.

Members of the Darwin Tree of Life Consortium are listed here: https://doi.org/10.5281/zenodo.4783558.
