# Supplementary material for: The genome sequence of spotted medick, Medicago arabica (L.) Huds. (Fabaceae)
Source: Wellcome Open Res. 2024 Nov 4;9:116. Originally published 2024 Mar 1. [Version 2] doi: 10.12688/wellcomeopenres.20996.2 (PMC11574327; doi:10.12688/wellcomeopenres.20996.2)
Supplement: Peer review history

# Reviewer report: Reviewer response for version 2

**DOI:** 10.21956/wellcomeopenres.25757.r112320

**Published:** 2024-11-28

**Authors:** Thomas Brazier

**Affiliations:** 1 University of Rennes, Rennes, France

This Data Note presents the first genome assembly for the spotted medick, *Medicago arabica*. This new reference genome assembly is chromosome-scale, with 8 chromosomes corresponding to the expected haploid chromosome number and the mitochondrial and plastid genomes.

 The ecology and genomics of the spotted medick are well described, and the importance of the study system is clearly stated.

 DNA extraction followed publicly available protocols from the Wellcome Sanger Institute and sequencing was done according to manufacturer protocols (Pacbio HiFi, 10X Genomics read cloud, Hi-C). Genome assembly was performed with state-of-the-art bioinformatic tools with rigorous QC and manual curation and evaluation. Globally the material and methods were clearly presented.

 The genome assembly is technically sound, and the QC is complete and appropriate. The final assembly covers 515 Mb of the 610 Mb estimated using flow cytometry (1C-value) and shows a high completeness. The results are sufficiently clear and detailed to evaluate that it is a good-quality genome assembly that will be an important reference genome for *Medicago* species.

 I just have a few minor comments, mostly typos:

 In the title, the 'ca' at the end of *Medicago arabica* is not italicised.

 In the 'Background' section, the sentence "Like many other *Medicago* species, spotted medick is rich in a variety of saponins with potential for use as antimicrobial compounds in agriculture and medicine (e.g. Avato et al., 2006; Bialy et al., 2004; Jarecka et al., 2008; Tava et al., 2009)." is repeated in two consecutive paragraphs.

 In the 'Genome Annotation Report' section, in the sentence "The Medicago arabica genome assembly (GCA_946800305.1) was annotated at the European Bioinformatics Institute (EBI)", *Medicago arabica* is not italicised.

 In the sentence "The average transcript length is 2,758.62", please add the unit "2,758.62 bp"

 Table 3. The gEVAL version is marked as 'N/A' since the website is not versioned. Maybe report the date (month/year) at which it was accessed in replacement of a proper version number.

 In the 'Genome annotation, curation and evaluation' section, you could use 'MercuryFK' as on the official website instead of 'Mercury. FK'

 Figure 5. The Hi-C contact map lacks axis ticks and labels.

Are sufficient details of methods and materials provided to allow replication by others?

Yes

Is the rationale for creating the dataset(s) clearly described?

Yes

Are the datasets clearly presented in a useable and accessible format?

Yes

Are the protocols appropriate and is the work technically sound?

Yes

Reviewer Expertise:

Plant comparative genomics and population genomics

I confirm that I have read this submission and believe that I have an appropriate level of expertise to confirm that it is of an acceptable scientific standard.

# Reviewer report: Reviewer response for version 2

**DOI:** 10.21956/wellcomeopenres.25757.r109992

**Published:** 2024-11-18

**Authors:** Yu Feng

**Affiliations:** 1 Chengdu Institute of Biology, Chinese Academy of Sciences, Sichuan, China

The manuscript reported high-quality a chrmosome scale assembly of the Medicago arabica genome, using Pacbio HiFi sequencing, 10X genomics and Hi-C library. There are still some points need to clarify:

 1. The Hi-C contact map lacks axis ticks and numbers. 

 2. The number of annotated coding genes are much less than the other two reported Medicago genomes. Is there only one tissue (leaf) were used in the RNA-seq to annotate the genome? Usually we use more tissues (e.g. flower, root, seed) to cover as many expressed genes as possible.

Are sufficient details of methods and materials provided to allow replication by others?

Yes

Is the rationale for creating the dataset(s) clearly described?

Yes

Are the datasets clearly presented in a useable and accessible format?

Yes

Are the protocols appropriate and is the work technically sound?

Partly

Reviewer Expertise:

Plant comparative genomics and population genomics.

I confirm that I have read this submission and believe that I have an appropriate level of expertise to confirm that it is of an acceptable scientific standard, however I have significant reservations, as outlined above.

# Reviewer report: Reviewer response for version 2

**DOI:** 10.21956/wellcomeopenres.25757.r109640

**Published:** 2024-11-15

**Authors:** Xiaolong Lyu

**Affiliations:** 1 College of Agriculture and Biotechnology, Zhejiang University, Hangzhou, China

This article is approved for indexing in current form.

Are sufficient details of methods and materials provided to allow replication by others?

Yes

Is the rationale for creating the dataset(s) clearly described?

Yes

Are the datasets clearly presented in a useable and accessible format?

Yes

Are the protocols appropriate and is the work technically sound?

Partly

Reviewer Expertise:

Plant science, genetics

I confirm that I have read this submission and believe that I have an appropriate level of expertise to confirm that it is of an acceptable scientific standard.

# Reviewer report: Reviewer response for version 1

**DOI:** 10.21956/wellcomeopenres.23231.r95261

**Published:** 2024-09-11

**Authors:** Xiaolong Lyu

**Affiliations:** 1 College of Agriculture and Biotechnology, Zhejiang University, Hangzhou, China

Christenhusz et al. present the first high-quality genome assembly of *Medicago arabica* (the spotted medick; Tracheophyta; Magnoliopsida; Fabales; Fabaceae).The genome assembly of *Medicago arabica *has important implications for the biochemical pathways of saponin compound synthesis, comparative genomics of cultivars and wild relatives, and agricultural breeding. A few points need to consider:

 In this study, the mitochondrial and chloroplast genomes of this species were assembled from PacBio HiFi reads using the MBG software, with these reads mapped to the relevant genomes. According to the website https://github.com/maickrau/MBG, the MBG software has undergone multiple versions, but the specific version used in the study is not provided. Given that different versions of MBG software and their outputs can vary, it is recommended that the authors specify the exact version used.

 Although this is the first high-quality genome assembly of *Medicago arabica*, it lacks functional and structural annotations. To make the genome more accessible for a wider audience, it is recommended to include the corresponding gene annotations.

Are sufficient details of methods and materials provided to allow replication by others?

Yes

Is the rationale for creating the dataset(s) clearly described?

Yes

Are the datasets clearly presented in a useable and accessible format?

Yes

Are the protocols appropriate and is the work technically sound?

Partly

Reviewer Expertise:

Plant science, genetics

I confirm that I have read this submission and believe that I have an appropriate level of expertise to confirm that it is of an acceptable scientific standard, however I have significant reservations, as outlined above.

# Reviewer report: Reviewer response for version 1

**DOI:** 10.21956/wellcomeopenres.23231.r92783

**Published:** 2024-08-29

**Authors:** Marco Pessoa-Filho

**Affiliations:** 1 Embrapa Recursos Geneticos e Biotecnologia, Brazilian Agricultural Research Corporation, Brasília, DF, Brazil

The data note describes the assembly of the spotted medick genome ( *Medicago arabica*), a species of the Fabaceae family native to the Mediterranean region and related to alfalfa and the bur clover.

 PacBio HiFi reads were obtained (44-fold coverage) and used for assembly with hifiasm. 10X genomics read clouds (86-fold coverage) were used for polishing. Hi-C reads generated with the Arima2 kit were used to obtain chromosome scale scaffolds with SALSA2. Manual curation was carried with gEVAL, HiGlass and PretextView. Merqury was used to assess k-mer completeness and QV consensus quality. RNAseq data was obtained from leaf tissue, but annotation was not reported in the data note.

 Raw data and assembly were deposited in INSDC databases and are publicly available.

 The rationale for creating the dataset was clearly described. Protocols were appropriate and the work is technically sound. The datasets are clearly presented in a usable and accessible format.

 More details on genome assembly, curation and evaluation could be provided in the Materials and Methods and would enrich the data note and allow its replication:

 1) Was Hifiasm run with default parameters?

 2) Considering that Hi-C data was available, was it used as input to Hifiasm for phasing?

 3) What output was further used in the pipeline for curation and scaffolding? Was it the p_ctg? The hap1_ctg?

 4) Considering that hifiasm already includes purging of haplotype duplications, why was purge_dups used? Was hifiasm run without haplotype duplication removal?

 5) More details on the polishing step must be provided. Considering HiFi reads low error rates, how much of an improvement is seen in the final assembly by running this step?

 6) What dataset was used as input to Merqury along with the assembly to assess k-mer completeness and QV consensus quality values?

 7) Table 3 lists MerquryFK, not the original Merqury. This should be mentioned in the text.

 8) Table 3 lists MitoHiFI but the text mentions MBG for mitochondrial and chloroplast genome assemblies. Which one was used?

Are sufficient details of methods and materials provided to allow replication by others?

Partly

Is the rationale for creating the dataset(s) clearly described?

Yes

Are the datasets clearly presented in a useable and accessible format?

Yes

Are the protocols appropriate and is the work technically sound?

Yes

Reviewer Expertise:

Plant genomics, bioinformatics

I confirm that I have read this submission and believe that I have an appropriate level of expertise to confirm that it is of an acceptable scientific standard, however I have significant reservations, as outlined above.
